# Future perspectives on swine viral vaccines: where are we headed?

**DOI:** 10.1186/s40813-020-00179-7

**Published:** 2021-01-04

**Authors:** Tanja Opriessnig, Ashley A. Mattei, Anbu K. Karuppannan, Patrick G. Halbur

**Affiliations:** 1 The Roslin Institute and The Royal (Dick) School of Veterinary Studies, University of Edinburgh, Midlothian, UK; 2 Department of Veterinary Diagnostic and Production Animal Medicine, College of Veterinary Medicine, Iowa State University, Ames, Iowa USA; 3 Vaccine Research Centre-Viral Vaccines, Centre for Animal Health Studies, Tamil Nadu Veterinary and Animal Sciences University, Chennai, India

**Keywords:** Vaccines, Review, Viruses, pigs

## Abstract

Deliberate infection of humans with smallpox, also known as variolation, was a common practice in Asia and dates back to the fifteenth century. The world’s first human vaccination was administered in 1796 by Edward Jenner, a British physician. One of the first pig vaccines, which targeted the bacterium *Erysipelothrix rhusiopathiae,* was introduced in 1883 in France by Louis Pasteur. Since then vaccination has become an essential part of pig production, and viral vaccines in particular are essential tools for pig producers and veterinarians to manage pig herd health. Traditionally, viral vaccines for pigs are either based on attenuated-live virus strains or inactivated viral antigens. With the advent of genomic sequencing and molecular engineering, novel vaccine strategies and tools, including subunit and nucleic acid vaccines, became available and are being increasingly used in pigs. This review aims to summarize recent trends and technologies available for the production and use of vaccines targeting pig viruses.

## Background

A vaccine is a substance used to provide immunity against a disease, by stimulating the production of antibodies, developing cellular immunity or both. It can be prepared from the causative agent of a disease, its products, or a synthetic substitute treated to act as an antigen without inducing the disease [1]. The origins of vaccinology, known as “variolation”, date at least back to the fifteenth century in China [2]. Variolation is a type of inoculation through which people were exposed to smallpox (variola) by blowing dried smallpox scabs into their nose (https://www.nlm.nih.gov/exhibition/smallpox/sp_variolation.html), and was introduced to Britain and New England early in the eighteenth century [3].

The term “vaccine” was created by Edward Jenner, an English physician working on smallpox in humans, it is thought to be derived from the Latin word “vacca” for cow and the cowpox virus administered to humans was designated as “vaccinia” [4]. This effort led to the first available vaccine for humans in 1796 [4]. Almost 100 years later, Louis Pasteur used the term vaccine in 1881 for immunizations directed at other diseases besides smallpox [5]. He also investigated mechanisms for attenuation and inactivation of microbes [5]. As a result, one of the first vaccines for pigs was introduced in France in response to severe disease outbreaks with high pig morbidity and mortality due to erysipelas [6]. This pig vaccine consisted of an initial exposure to attenuated-live bacteria derived from passaging in rabbits (also known as lapinization), followed by inoculation of unaltered live virulent bacteria 12 days later [6].

Early efforts in large-scale controlled processes and industrialization of veterinary vaccine production began between 1930 and 1940 in Germany to produce foot and mouth disease virus (FMDV) vaccine antigen [7]. Both bacterial and viral vaccines commonly used the attenuated-live or the inactivated approaches to allow for consistency and larger commercial production, ultimately resulting in wider distribution. Production and management changes, including increasing herd size and housing, genetic improvements of the pig itself, nutritional changes, generation of specific-pathogen free herds and other changes were introduced to pig production towards the end of the last century. These changes also opened up avenues for easier and more rapid virus spread, resulting in high morbidity and mortality in some pig production systems. Hence, the need to protect pigs from viruses via vaccination has become critical for many pig producers. Both attenuated-live and inactivated vaccine approaches are technologies from more than a century ago, yet they have remained popular in today’s pig production industry.

Many of the vaccines used today are far from perfect. An ideal vaccine should produce “sterilizing immunity” in the vaccinated individual or animal, i.e., exposure to the pathogen should not result in any infection [8]. However, the many subtleties of the phenomenon of evolution have given pathogens an upper hand against vaccines for several diseases. Pathogen species are often highly variable and commonly differentiated as serotypes/serovars, genotypes, low/medium/high virulent isolates, among other terms. Furthermore, they continue to evolve in the face of vaccination. Even within a species, pathogens vary significantly in their pathogenicity and virulence. Pathogens also vary in their tissue of preference for replication and their mode of transmission. Examples include simple secretion into the environment versus very specific transmission through coitus, across the placental barrier, or via saliva through biting. As the timeline of all of these processes is different, this is enumerated as the basic reproduction number (R_0_) which indicates how contagious an infectious disease is; an outbreak is expected to continue if R_0_ has a value > 1 and to end if R_0_ is < 1 [9]. This broadly depends on the period of shedding of the pathogen from the infected animal, mode of transmission of the infection, and herd density/contact rate between susceptible individuals [9]. In addition to pathogen-specific factors, the host immunogenetics also play a role in the development of immunity and resistance to infection. Hence, developing vaccines is unique for every infection or pathogen. In addition, a less than perfect vaccine, which does not elicit a sterile immunity, may still be useful in mitigating an infection at the individual pig level and limit its spread to the rest of the herd by reducing the shedding of the pathogen and enhancing a pig’s resistance to initiation of infection.

This review aims to provide a summary on viral vaccines in pigs with an outlook on new developments that may become important in the near future.

### Source of virus vaccines

#### Commercial vaccines

In general, the virus contained in a vaccine candidate is sourced from the overall virus population of a larger geographic area with the purpose to protect as many pigs as possible. The sourcing and selection process can take months to years and is principally influenced by the ability of the vaccine candidate to induce a response that broadly neutralizes prevailing variants (serotypes, genotypes, etc.) in the region where the vaccine is to be used. This is followed by government-stipulated safety and efficacy trials in experimentally-infected pigs and in field trials. If all set requirements are fulfilled, eventually the vaccine is commercialized, licensed for a certain area and then becomes available for use. Some vaccine viruses may also be used on a global basis if they match and protect against most virus variants present and if regulatory compliance in each of the countries has been fulfilled. For example, the source of one of the major attenuated porcine reproductive and respiratory syndrome virus (PRRSV) vaccines, Ingelvac PRRS® MLV, Boehringer Ingelheim Vetmedica, was the US field strain VR2332 isolated in Minnesota, USA in 1992 [10]. This vaccine first became available in 1994 and continues to be widely used on a global basis [11]. Most of the currently used inactivated porcine parvovirus (PPV) vaccines are based on strain NADL2 [12] which was first described in 1976 [13]. A novel subunit PPV vaccine (ReproCyc® ParvoFLEX, Boehringer Ingelheim) came on the European market in 2018 and is based on strain 27a, first isolated in 2001 [14].

#### Autogenous vaccines

For some viruses, especially those that mutate frequently (i.e. RNA viruses) and where several diverse genotypes exist, commercial vaccines, which incorporate a “representative vaccine candidate”, may not always provide sufficient protection. In this case, autogenous vaccines can be used. Autogenous vaccines utilize local farm-specific strains that are isolated and propagated in specialized labs before a final vaccine preparation is sent back to the farm of origin where it is used to protect the pig herd. Safety and efficacy trials are not typically required or completed. This approach is commonly used for viruses such as PRRSV, where there are many different virus types and vaccine protection is considered to be correlated to its closeness to the farm-specific virus. Due to biosecurity risks, autogenous vaccines are only allowed to be used on the farm or within the production system from where the virus originated. In general, guidelines on production and use of autogenous vaccines vary from country to country and commonly there are certified autogenous vaccine production sites available.

#### Individualized vaccines

This refers to vaccines that to the authors’ knowledge are not available yet but are designed for pigs with a specific genetic background. For example, different pig breeds such as Pietrain, Large White, Landrace or others, and even lineages within a breed, may vary in their response to a given vaccine and investigating this may offer new clues on decreased vaccine efficacy on certain farms. Although there is not an exhaustive knowledge base built on the immunogenetics of swine, it is known that variations in immune response occur due to swine major histocompatibility complex (MHC) polymorphisms [15]. However, differences in MHC genes are just one aspect of immunogenetic variations and more remains to be unravelled. It is already known that in humans females have a better immune response compared to males [16, 17]. It is presently unknown if this observation holds true in pigs or other animal species. The accumulation of knowledge in the various disciplines of vaccinology may phase out the era of “one size fits all” vaccines in favour of products individually tailored for a specific population of pigs.

## Composition

There are several types of vaccines commonly used in pigs. Broadly, these can be categorized into whole pathogen, subunit and nucleic acid-based vaccines (Table 1). Moreover, vaccines can also be monovalent (protection against a single virus strain) or multivalent/polyvalent (protection against two or more strains of the same virus or two or more different viruses). Vectored vaccines in particular have great potential to protect against more than one strain or virus. An excellent review has been recently published on this topic [18].

**Table 1:** Types of vaccines used in pigs

Main vaccine category	Subtypes
***Whole pathogen***	• Attenuated-live
	• Inactivated (killed)
	• Chimeric (attenuated-live or inactivated)
***Subunit or vector system based***^***a***^	• Prokaryotic (unicellular organisms without a distinct nucleus) ○*Escherichia coli*, *Bacillus subtilis* • Eukaryotic (unicellular or multicellular organisms with an enveloped nucleus) ○Yeast, mammalian cells, baculovirus expression in insect cells, plants, transgenic animals • Live mammalian virus vectors (replicating or replication deficient)
***Nucleic acid based***	• DNA plasmid(s) • mRNA

^*a*^Virus-like particles (VLPs) are often produced

### Whole pathogen vaccines

#### Attenuated-live whole pathogen vaccines

Attenuated-live vaccines are typically obtained by serial passaging viral strains either in pigs, in heterologous species such as rabbits (lapinization), or in cell culture. The virus stock is regularly checked during the serial passage until inoculated pigs no longer display clinical signs. This attenuated virus vaccine stock is then used for vaccine production. Attenuated-live vaccines, which mimic the process of natural infection but are not pathogenic, elicit strong immune responses that can confer life-long immunity after only one or two doses. Especially for RNA viruses, which include PRRSV and influenza A virus (IAV) among other viruses, the immune response is greatly improved if it is triggered by a live virus. Attenuated-live vaccines are relatively easy to create for certain viruses but also have major disadvantages, including safety (Table 2). Attenuated-live vaccines are commonly used for control of PRRSV [11] and are widely available on a commercial basis (Table 3). However, as there are two main PRRSV species and many subtypes within each species [19, 20], protection is commonly only efficient with homologous vaccine virus strains and requires genomic sequencing of the farm specific PRRSV strain first [21]. Regardless, genomic homology of the PRRSV open reading frame (ORF) 5 sequence of attenuated vaccines is not always predictive of their efficacy [22]. Safety issues with PRRSV attenuated vaccines are not uncommon [23, 24]. A recently documented incident demonstrates the recombination of two species of PRRSV vaccine strains associated with severe disease outbreaks in Danish pigs in 2020 [25]. A bivalent attenuated-live IAV vaccine entered the US market in 2017 and is licensed for piglets as young as 1 day old. In experimental trials this vaccine showed significant reduction of nasal IAV shedding when pigs were challenged 12 weeks after vaccination [26]. Recently, evidence of re-assortment of the live attenuated IAV vaccine virus with endemic field strains circulating in the US was detected [27]. The report raises concerns over the safety of this particular vaccine.

**Table 2:** Simplified advantages and disadvantages of traditionally used, widely available viral attenuated-live and inactivated vaccine types in pigs

	Attenuated-live	Inactivated
***Administration routes***	(IM), oral, intranasal, intradermal	IM, intradermal
***Adjuvant***	None	Required
***Number of doses***	One dose	Often two doses
***Humoral immunity***	IgG	IgG
***Secretory immunity***	IgA	–
***Cellular immunity***	Yes	–
***Long-term immunity***	Yes	–
***Reversion to virulence or recombination***	Yes	Not possible
***Storage***	Heat-labile^*a*^	Stable^*b*^
***Transmission among pigs***	Yes	No
***Production cost***	Low *(involves only culture, bottling and lyophilization or freezing)*	High *(requires a high antigen content, addition of adjuvant and other excipients)*
***Time until a vaccine is available***	Months to years	Months

^*a*^Needs to be stored at −80 °C or lyophilized. Cannot be stored once opened

^*b*^Storage at room temperature or at + 4 °C (never frozen) for longer periods; can be used for some time after opening a bottle. Phase separation of the aqueous and oil phases in the vaccine formulation may be observed in some vaccines, which can be corrected by vigorous shaking of the vaccine container

**Table 3:** Examples of commonly used viral pig vaccines available in European and North American regions

	Vaccine type	Example viruses				
		***PRRSV***	***IAV***	***PCV2***	***PPV***	***PEDV***
**Whole pathogen**	***Attenuated-live***	Ingelvac® PRRS MLV (BIVI) Ingelvac® PRRS ATP (BIVI) ReproCyc® PRRS EU (BIVI) Porcilis® PRRS (MSD) Fostera® PRRS (Zoetis) Suvaxyn® PRRS MLV (Zoetis) Prime Pac® PRRS RR (Merck) Prevacent® PRRS (Elanco) UNISTRAIN® PRRS (HIPRA) AMERVAC® PRRS (HIPRA)	Ingelvac Provenza® (BIVI)			
	***Inactivated***	Progressis® (Ceva) SUIPRAVAC® PRRS (Hipra)	Suvaxyn® SIV (Zoetis) FluSure® Pandemic (Zoetis) FluSure XP® (Zoetis) Respiporc FLUpan® H1N1 (Ceva) Respiporc FLU3® (Ceva) GRIPORK® (HIPRA)	Circovac® (Ceva) Suigen® PCV2 (Virbac)	Parvovax® (Ceva) Suvaxyn® Parvo (Zoetis) FarrowSure® Gold (Zoetis) PARVOSENG® (HIPRA) Parvo Shield® (Elanco) Porcilis® Parvo (MSD)	Porcine epidemic diarrhea vaccine (Zoetis)
	***Chimeric***	PRRSGard® (Pharmgate; attenuated-live)		Fostera® PCV (Zoetis; inactivated) Suvaxyn® Circo (Zoetis; inactivated)		
**Subunit**	***Baculovirus expression in insect cells***			Ingelvac CircoFLEX® (BIVI) Porcilis® PCV (MSD) Circumvent® PCV (Merck) CircoGard® (Pharmgate)	Reprocyc® ParvoFLEX (BIVI)	
	***Mycoplasma hyopneumoniae expression vector***			MHYOSPHERE®PCV ID (HIPRA)		
	***Replication-deficient viruses***	SEQUIVITY® product line (Merck; USA) can be customized to any virus				

A novel approach to rapidly attenuate viruses includes computer-aided codon pair deoptimization, also known as synthetic attenuated virus engineering (SAVE) approach [28]. For pigs, this approach has been experimentally investigated with PRRSV and indeed codon pair deoptimization of the gene encoding the major envelope GP5 protein resulted in attenuation of the virus [29]. In a subsequent challenge study, the previously constructed SAVE PRRSV vaccine virus was effective in protecting pigs against homologous virus challenge [30]. These results are promising as overall production of a deoptimized vaccine virus may only take weeks to month. Additionally, in contrast to traditional attenuated viruses, which have an inherent risk of reversion to virulence, the deoptimization during the SAVE approach induces several minor changes which make a reversion to virulence unlikely [29].

#### Inactivated whole pathogen vaccines

These vaccines consist of entire pathogens that have been inactivated or killed so that they cannot cause disease in vaccinated pigs. Vaccines in this category are produced by inactivating the pathogen with chemical (commonly formaldehyde; beta-propiolactone or binary ethylenimine) or physical (thermal such as heat or radiation) treatment. Inactivated vaccines emerged for use in pigs shortly after World War II and several adaptions and improvements have been introduced over time. Advantages and disadvantages of inactivated vaccines are listed in Table 2 and compared with attenuated-live vaccines. A pig virus that is commonly controlled by use of inactivated vaccines is IAV [31]. This may be in part due to the perceived risk of live vaccines giving way to reassortant viruses or reversion to virulence [27]. Any new IAV variant (recombination, re-assortment) could be detrimental to pigs but also other species including humans due to its cross-species tropism. A recent example of recombination of IAV portions from different species resulted in the pH1N1 pandemic during 2009 [32].

#### Chimeric whole pathogen vaccines

Genetic engineering techniques have enabled the creation of chimeric viruses, which contain genetic information from and display biological properties of two different parent viruses. Such vaccines represent a whole fused pathogen and often the fusion of two virus portions results in natural attenuation and reduction of replication. These attenuated-live chimeric viruses can be further inactivated for additional safety. Alternatively, chimeric viruses may not be viable and hence are considered inactive. An example is a chimeric virus used to vaccinate pigs against porcine circovirus type 2 (PCV2). While PCV2, discovered in 1998, is pathogenic, porcine circovirus type 1 (PCV1), discovered prior to PCV2 in the 1970s, is non-pathogenic [33]. A chimeric infectious clone was created in 2002 by fusing PCV1 ORF1 (responsible for viral replication) with PCV2 ORF2 (encodes the capsid protein against which antibodies are generated) [34]. The resulting PCV1–2 chimeric virus was shown to replicate in pigs at a low level without causing disease or lesions; however, it generated a strong humoral immune response to PCV2 [34]. In subsequent studies the attenuated PCV1–2 was found safe for use in pigs and able to protect growing pigs from the consequences of PCV2 challenge [35, 36] and also pregnant sows [37]. The final vaccine preparation that entered the market as a licensed vaccine contained an inactivated version of the PCV1–2 [37]. With expanding knowledge on PCV2, several genotypes were identified of which PCV2a, PCV2b and PCV2d appear to be of greatest importance [38]. This vaccine has now been updated to contain two different PCV1–2 types, one based on ORF2 of PCV2a and one based on ORF2 of PCV2b (https://www.zoetisus.com/news-and-media/zoetis-introduces-u.s.-pork-industry_s-first-vaccine-with-two-pcv2-genotypes.aspx). A slightly different approach in addition to the chimeric PCV1–2 clone was introduced in 2016 [39]. In brief, the capsid gene (ORF2) of available PCV2 genotypes was subjected to deoxyribonucleic acid (DNA) shuffling resulting in a capsid gene that contained portions of different PCV2 genotypes. This so generated capsid sequence was inserted in the PCV1 backbone as described above. This chimeric virus induced protective immunity against PCV2b and PCV2d under experimental conditions [39]. A chimeric full-length PRRSV clone was generated by inserting shuffled structural genes (ORFs 3–6) from six heterologous PRRSV strains into the backbone of PRRSV strain VR2385. This chimeric PRRSV virus conferred an enhanced cross-protection in vaccinated pigs when challenged with the heterologous virus strain NADC20 or the contemporary heterologous strain RFLP 1-7-4 [40].

### Subunit vaccines

This type of vaccine contains viral components, also known as antigens, capable of stimulating the immune system. Antigens are commonly expressed using prokaryotes (*Escherichia coli, Bacillus subtilis*), eukaryotes (yeast, mammalian cells, baculovirus in insect cells, plants, and animals), or mammalian viruses including replicating or replication deficient variants. In general, the subunit vaccine design can make vaccines safer as well as production and quality control between batches easier [41] compared to live attenuated viruses or inactivated viruses. However, incorporation of adjuvants to elicit a strong protective immune response is often essential. Subunit vaccines can be administered as crude material that includes the expression vector, sometimes with adjuvant properties, or as purified proteins. Crude material for instance was used in the early days of PCV2 vaccination and an ELISA detecting baculovirus antibodies was offered at certain diagnostic laboratories including at Iowa State University to confirm successful vaccination of pigs (TO personal observation). Another example is the BAYOVAC CSF E2 vaccine for classical swine fever (Bayer AG, Germany), which in 1998 contained the E2 protein of the virus along with chemically (BEI) inactivated baculovirus (https://www.ema.europa.eu/en/documents/procedural-steps/advasure-epar-procedural-steps-taken-authorisation_en.pdf). It is possible that the included antigens alone are not sufficient to induce adequate long-term immunity; however, including only the essential antigens in a vaccine can minimize any potential side effects.

#### Prokaryotic vectors (i.e. primarily unicellular organisms without a distinct nucleus)

The *E. coli* vector has a fast growth rate, an overall low cost and high protein yield. Disadvantages include the lack of eukaryotic co- and post-translational modifications, proteins may often be insoluble, and the codon usage is different from eukaryotes. Specifically engineered bacteria, able to induce specific post-translational modifications, are now occasionally used for a unique protein of interest but the process is difficult [42]. Several experimental PCV2 vaccines have been described using the *E. coli* vector [43–45]. In addition, an *E. coli* -expressed PRRSV chimeric protein induced a specific immunoglobulin G response in vaccinated pigs [46]. The *B. subtilis* vector has been experimentally used in pigs as an oral vaccine expressing the PCV2 capsid protein [47]. A mucosal response was seen when piglets were vaccinated orally with the porcine epidemic diarrhea virus (PEDV) COE antigen, which is a collagenase-digested fragment of the spike protein, expressed by *B. subtilis* [48]. Advantages of *B. subtilis* include that this vector can be used for mucosal immunization via a non-invasive needle-free route, as it is considered a temperature resistant immunogen [49].

#### Eukaryotic vectors (i.e. unicellular or multicellular organisms with an enveloped nucleus)

##### Yeast

Advantages of yeast vectors are a fast growth rate, a high protein yield, low production cost, and good protein folding. Despite these advantages this vector is not commonly used in pig vaccinology. Commonly used yeast vectors include *Saccharomyces cerevisiae, Schizosaccharomyces pombe, Saccharomyces boulardii, Hansenula polymorpha, Pichia pastoris, Candida boldmu, Kluyveromyces lacti, Yarrowia lipolytica*, and others. Yeast vectors in pigs have been suggested for vaccines against PEDV. Specially, under experimental conditions, a whole yeast vaccine expressing the PEDV S1 protein induced a high PEDV IgA response in pigs when administered orally [50]. Another example of this expression platform in pigs is the production of the ectodomain of the immunologically relevant hemagglutinin-neuraminidase glycoprotein of porcine rubulavirus, which induced neutralizing antibodies in mice [51].

##### Mammalian cells

Mammalian cells are naturally fitted for the production and secretion of complex molecules with precise glycosylation. However, this system has not yet been adopted for common use in pig vaccine production. In general, the speed of production is low, the cost is high, and there is also the potential for endogenous virus contamination [52]. However, clear advantages of this expression system include that there is a medium-to-high scale-up capacity, the system overall can reach high and robust productivity of secreted proteins in serum-free medium, and mammalian cells have the ability to perform complex post-transcriptional modifications. The two most common cell lines employed are the Chinese hamster ovary (CHO) and the human embryonic kidney 293 (HEK-293) cell lines [52]. A recent study compared the ability of yeast, insect and mammalian cells to produce a PEDV S1 vaccine protein [53]. Of the three eukaryotic expression systems tested, HEK-293 T cells gave the highest yield of protein (N-glycosylated). Vaccination of a sow resulted in induction of S1-specific IgG and IgA that were passively transferred to the suckling piglets. High virus neutralization titres were measured in the serum of the vaccinated sow and its piglets [53]. In another example, HEK-293 cells were used to express the classical swine fever virus (CSFV) E2 glycoprotein [54]. Similarly, CSFV E2 proteins have also been successfully produced in CHO cells [55].

##### Baculovirus vector in insect cells

This system results in good protein folding; however, the protein yield is lower than with other methods and hence raises production costs. The baculovirus vector has been used for numerous experimental vaccines and is now used in the production of several commercial pig vaccines (Table 3). Among many others, additional examples where the baculovirus vector has been used successfully under experimental conditions include pseudorabies virus (PRV) (glycoprotein D) [56], hepatitis E virus (HEV) [57] or CSFV [58].

##### Plants

Examples of plant-expressed vaccines include CSFV, for which the structural glycoprotein E2 is commonly used [59, 60]. For example, when using the *Nicotiana benthamiana* plant expression system, purified recombinant E2 protein generated high titers of neutralizing antibodies in pigs and mice when injected intramuscularly [60]. Furthermore, transplastomic tobacco was used to produce a subunit vaccine candidate against PEDV [61]. Interestingly, the plant biomass matrix was shown to delay degradation of the chloroplast-produced rFaeGntd/dsc in gastrointestinal conditions, suggesting its possible use as an oral vaccination strategy [61]. Similarly, embryogenic cells of banana plants have been transformed with the ORF5 gene of PRRSV using *Agrobacterium*-mediated transformation [62]. Pigs were orally immunized with recombinant GP5 protein by feeding them with transgenic banana leaves three times at 2-week intervals and were then challenged with PRRSV at 7 weeks post initial immunization. These orally vaccinated pigs had increased anti-PRRSV IgG and IgA antibodies, lower viremia and tissue viral loads compared to non-vaccinated pigs [62]. There have also been reports of PRRSV expression in tobacco [63] and potato plants [64]. Advantages of the plant expression system versus the baculovirus/insect cell system are the potential scalability as well as the lower cost of production and immunogenicity [59].

##### Transgenic animals

Producing recombinant proteins in animal bioreactors could present an excellent tool for vaccine production [65]. For instance, a certain vaccine protein could be excreted in the milk of dairy cattle via generation of transgenic animals. However, negative aspects include the long time between the production of such a transgenic cow and its first lactation cycle, which additionally is intermittent in nature. The use of transgenic chicken eggs for large scale recombinant protein production appears to be a better avenue; however, so far this system has mainly been used for the production of specific antibodies by vaccinating the hen to provide passively acquired immunity to other species [65]. In pigs, a specific example is their passive protection from FMDV infection and transmission by immunization with Llama single-domain antibody fragments [66].

##### Virus-like particles

VLPs are self-assembling, non-replicating and non-pathogenic, highly organized, supramolecular multi-protein nanoparticles (ranging from 20 to 100 nm) that can be formed from the minimal spontaneous self-assembly of one or more viral structural capsid proteins, giving empty viral particles devoid of genetic material [67]. Hence, they prompt an immune response similar to that elicited by the natural virus, but VLPs are non-infectious because they do not contain the genetic material the virus needs to replicate inside cells. VLP assembly may also take place inside the host cell through self-assembly. VLPs are usually produced in bacteria, yeast, or the baculovirus/insect cell system [68]. Several baculovirus-expressed commercial PCV2 vaccines have been shown to contain a certain percentage of VLPs [69]. Recently, an efficient application of a baculovirus-silkworm larvae expression system for PCV2 VLP production has been described [70]. Efficient production of PCV2 VLPs was reported using the non-conventional yeast species *Kluyveromyces marxianus* [71]. Foot-and-mouth disease virus (FMDV) VLPs have been suggested as a non-replicating vaccine candidate [72]. Specifically, VLPs composed entirely of FMDV capsid proteins were simultaneously produced as small ubiquitin-like modifier (SUMO) fusion proteins by an improved SUMO fusion protein system in *E. coli.* Intramuscular immunization of pigs with the FMDV VLPs induced a protective immune response against homologous FMDV challenge [72]. Finally, several studies reported the generation of *E. coli*-derived PCV2 VLPs [73, 74]. Of note, self-assembling proteins could coincide with the actual antigen for vaccination or could be carriers for other antigens expressed as fusion proteins. An example of the latter is the use of Hepatitis B virus core antigen as a carrier protein for the conserved protective epitopes of PRRSV [75]. This hybrid VLP system offers low cost production and is safe [75].

#### Live mammalian viral vectors

##### Replicating mammalian viruses

PRRSV has been proposed as a live viral vector by using an attenuated strain (DS722) to express protective antigens from IAV and PCV2 [76]. A vaccination and challenge study in 48 pigs revealed that the DS722-SIV-PCV2-vaccinated pigs had significantly reduced lung lesions and viral RNA loads when challenged with PRRSV. Upon challenge with PCV2, the vaccinated pigs had partially reduced lymphoid lesions and viral DNA loads, and when challenged with IAV the vaccinated pigs had significantly reduced acute respiratory sign scores [76].

##### Replication-deficient viruses

These viral vectors are deficient of viral functions essential for replication and assembly of progeny virus particles [77]. These viruses are propagated in cell lines which complement the missing viral gene products; hence gene expression takes place in infected cells, but no virus progeny is produced. Examples of replication deficient viruses include poxvirus, adenovirus and alphavirus species. The modified vaccinia Ankara (MVA) strain is an attenuated vaccine based on poxvirus [78]. A subset of African swine fever virus (ASFV) antigens were purified from HEK cells and produced in MVA viral vectors and evaluated in pigs using a prime-boost concept [79]. This resulted in induction of ASFV specific antibody and T-cell responses demonstrating the feasibility of generating a safe and immunogenic vaccine against this devastating pig disease [79]. Adenovirus vectors have been experimentally used in pigs to protect them against PRRSV [80] or PEDV in the form of a mucosal vaccine [81]. The adenovirus platform was also recently used to develop PRRSV species 2 subunit vaccines by expressing swine leukocyte antigen (SLA) class I and class II allele-specific antigens [82]. Specifically, two PRRSV poly-T-cell epitope peptides each specific to SLA I or SLA II were used. However, in the pig challenge study these vaccines provided little improvement over non-vaccinated pigs highlighting the challenges in developing effective PRRSV subunit vaccines [82]. Alphaviruses have a broad host cell range, a tropism for dendritic and monocytic cells and hence can stimulate innate immune responses [77]. In addition, in most hosts including pigs there is no pre-existing immunity. Alphavirus vectors resemble yet another single stranded replicating RNA vector and demonstrate a high level of transient heterologous gene expression in-vivo and in-vitro [83]. The most commonly used encapsulated alphaviruses include Semliki Forest virus, Sindbis virus and Venezuelan equine encephalitis virus [83]. The last one has been licensed in the US as a vector for autogenous vaccine production (SEQUIVITY® product line; Merck, USA) and many such vaccines are now being used in the field. The alphavirus platform has previously been successfully employed to protect pigs from PCV2d challenge under experimental conditions [84]. The alphavirus vector was also used for cluster IV H3N2 IAV vaccine production [85].

### Nucleic acid based vaccines

#### DNA plasmid vaccines

These typically comprise a small circular piece of episomal DNA derived from prokaryotes, called a plasmid, that is used as a vector to carry a gene(s) encoding proteins from the pathogen of interest [86]. This is injected directly into the muscle of the recipient. The muscle cells take up the DNA, and the encoded protein antigen is expressed in the cells, leading to both humoral and cell-mediated immune responses. Local dendritic cells play an important role in the development of antigenic responses to DNA vaccines. As only a single microbial gene or DNA encoding a set of antigenic peptides is used, it is safe and does not carry any risk of active infection [86]. The manufacturing process for DNA plasmid vaccines is well-established, allowing experimental vaccines to be quickly developed to address emerging or re-emerging infectious diseases. DNA plasmid vaccines have been used under experimental conditions for IAV protection and resulted in improved cellular immunity compared to traditional inactivated vaccines [87]. Recently, a DNA vaccine using a mosaic vaccine approach has been described [88]*.* Mosaic vaccines are prepared for viruses that have many different strains with limited cross-protection. A mosaic vaccine combines pieces of different strains with the goal to evoke a broad immune response. A PRRSV DNA vaccine was constructed with ORF5 PRRSV mosaic sequences, complexed to cationic liposomes, and administered to pigs using intradermal and intramuscular routes. This vaccine has been found to induce cellular immune responses against several PRRSV species 2 strains. In a subsequent challenge study, the vaccine was found to induce broad protection against heterologous PRRSV strains when administered twice to pigs [88]. In addition, a DNA vaccine based on conserved haemagglutinin (HA) peptides fused with flagellin, administered with a needle-free device, induced a strong immune response and rapidly cleared IAV from vaccinated pigs [89]. Maternally derived antibodies did not interfere with vaccination and there was a markedly increased mucosal IgA response in vaccinated pigs compared to non-vaccinated pigs [89].

#### mRNA vaccines

Vaccines based on messenger RNA (mRNA), an intermediary between DNA and protein, are also being developed. Recent technological advances have largely overcome issues with the instability of mRNA and the difficulty of delivering it into cells, and some mRNA vaccines have demonstrated encouraging early results. mRNA-based vaccines have important advantages over other vaccine approaches, including outstanding efficacy, safety, and the potential for rapid, inexpensive, and scalable production [90]. A rabies vaccine for pigs has been reported as an example of this vaccine platform [91]. In brief, an optimized non-replicating rabies virus glycoprotein encoding mRNA was used to induce potent neutralizing antibodies in domestic pigs. Virus neutralization titers which correlated with protection in adult and new-born pigs, were successfully induced, demonstrating the feasibility of a non-replicating mRNA rabies vaccine in pigs and highlighting the promises of mRNA vaccines for the prevention of infectious diseases [91]. Of note, to counter the current severe acute respiratory syndrome-coronavirus-2 (SARS-CoV-2) epidemic, several mRNA vaccines for human use have recently been licensed [92, 93].

### DIVA vaccines

It is often of interest to have vaccines available that can help differentiate infected from vaccinated animals, also known as DIVA or marker vaccines. DIVA vaccines induce an immune response which is different from that induced by natural infection. Two types of markers can be used: Negative markers (the vaccine has at least one antigenic protein less compared to the field strain) or positive markers (an antigenic protein is inserted in the vaccine) [94]. Overall, negative markers are preferred as they allow for identification of pigs infected with field virus which then can be removed [94]. The main advantage of DIVA vaccines and their accompanying tests is the possibility to distinguish between infected and vaccinated animals. Therefore, restrictions in place for infected animals can be reduced for vaccinated animals. In pigs, DIVA vaccines were first used for PRV also known as Aujesky’s disease. Most were based on recombinant deletion mutants that lack the gE envelope glycoprotein and thymidine kinase genes [95]. Many of these deletion mutants were detected in live-attenuated PRV vaccine strains in the mid 1980s [95]. A competitive enzyme immunoassay was concurrently developed [96] and the access to a marker vaccine and an assay to differentiate infected from vaccinated pigs facilitated the eventual eradication of PRV [97]. A commercial DIVA vaccine for CSFV, a baculovirus-expressed recombinant E2-subunit vaccine, is also available on the market [98].

### Vaccine administration

There are a variety of traditional, trending, and alternative vaccine administration routes that are used to vaccinate pigs (Table 4). In general, pig vaccine administration should be fast and pig, friendly, with as little stress as possible on the individual pigs and the people administering the product. Preferably, the mode of vaccination should not be labour intensive. Intramuscular injection is the most commonly used administration route. Recently, routes such as intradermal, intranasal or oral have at least been demonstrated to have the potential, in the future, to replace the traditional long-standing intramuscular vaccination route. Especially for intradermal applications, the dermis is an excellent site for vaccine delivery and rich in dendritic cells as well as lymph vessels which promote fast processing of incoming antigen (http://www.positiveaction.info/pdfs/stars/pt_MSD1-8.pdf). DNA vaccines appear to benefit from the intradermal route of administration [87, 89]. Several commercial health companies have started to produce their own, individual intradermal injection devices, which can be used on company-specific vaccine bottles, and are sold to producers as package deals. These devices either use needles or are needle-free. It has been demonstrated that needle-free intradermal vaccination reduces fear and pain reaction in gestating sows during vaccination [99]. As an example for the oral administration route, a recent study using a bread-based lyophilized C-strain of CSFV, tested on backyard pig farms, has provided encouraging results [100]. In an attempt to improve storage conditions for backyard farmers by avoiding the need for freezing, dog food, horse feed, pig feed, rice bran, and plain sliced bread were used as candidates for the vaccine base. Titres of the bread-based lyophilized CSFV vaccine were stable at around 3.67 log_10_ 50% tissue culture infectious dose (TCID_50_) per ml for 7 months at 4 °C. Pigs that orally received bread-based lyophilized CSFV vaccine showed seroconversion of over 90% at 14 days post vaccination. This study is also an example of how vaccine thermostability was improved to allow vaccine delivery to be less dependent on functioning cold chains [100].

**Table 4:** Overview of different vaccine administration routes. The subcutaneous route, as it is not commonly used, is not included

Route	Device	Immunity	Restraint of pigs	Average volume	Stress on pig	Pathogen transmission	Comments
***Intramuscular***	Needle	Systemic	Yes	1–2 ml	Yes	Yes	Risk of needle breakage and introduction of other infections through needle puncture is high. In addition, this method is labour intensive.
***Intradermal***	Needle or needle free device	Systemic	Yes	0.5 ml	Yes	Only if a needle is used	Special devices and training may be needed initially.
***Intranasal***	Syringe with or without adaptor	Mucosal	Yes	0.5-2 ml	Yes Yes	Minimal	Perhaps difficult in breeding animals; localized or mucosal immunity may be a possible benefit in vaccination against respiratory viruses.
***Oral***	Syringe, feed or water	Mucosal	No	Not applicable	No	No	Attenuated virus vaccines are needed. Pigs need to have access to the feed/water and need to be willing to eat or drink at vaccination. Water quality and feed ingredients may interfere.

The benefits of alternative routes may include induction of a strong localized/mucosal immune response, especially at the point of virus entry (i.e. respiratory system) [101, 102].

### Adjuvants

Adjuvants, pharmacological or immunological agents that improve the immune response of a vaccine, are integral components of most vaccines used today in pigs. Adjuvants are used to reduce the number of individual vaccine injections, which results in reduced labour, animal stress, risk of injuries to the pig (needle breaks, injuries due to catching the pig) and ultimately reduced cost for the producer [103]. They can be broadly divided into major groups based on their main component: oil emulsion, particulate antigen carrier, cytokines, pathogen associated molecular patterns, and saponins. Adjuvants are often specific for certain routes of vaccination. For example, while the same vaccine can be used orally, intranasally or intramuscularly, each of these routes may require a different route-specific adjuvant to enable optimal vaccine performance. Adjuvants have become valuable assets for commercial vaccine companies and are commonly protected by patents. A detailed review on adjuvants used in pigs including their mechanism of action has been published recently [103].

### Nanoscale/microscale carriers for delivery of vaccines

Nanoparticle-based technology has received a lot of attention lately. Nanocarriers, able to generate strong protection after a single dose, are used to present a virus vector, protein, subunit antigens or VLP to the immune system. There are several options for nanocarriers that can be used in nanovaccines including spores (bacterial), proteasomes (cell membrane based), exosomes (cellular), liposomes (lipid-based), virosomes (liposome and viral envelope protein), super fluids (biodegradable polymer), nanobeads (inert nanomaterial), VLPs (viral) and phages (viral, bacteria as targets) [104]. Most induce humoral and cellular immunity. At this point, commonly used nanocarriers in pigs are polymeric nanoparticles including polysaccharides, polyesters, chitosan and others. Their production, characterization, toxicology and ecotoxicology have been recently reviewed [105]. For instance, an experimental inactivated IAV vaccine was generated using chitosan and induced cell-mediated immunity in pigs vaccinated intranasally [106]. The IAV extracellular domain of M2 (M2e) antigen was inserted into the capsid protein of PCV2 and expressed in *E. coli* to form a self-assembled chimeric VLP nanovaccine [107]. High levels of M2e specific antibodies and PCV2 specific neutralizing antibodies could be induced in vaccinated pigs [107]. The immunogenicity and protective efficacy of a polyanhydride nanoparticle-based IAV vaccine, in which inactivated soluble antigen was co-encapsulated with a toll-like receptor 9 agonist, was tested in pigs challenged with IAV [108]. Results indicated that high mucosal humoral and cellular immune responses in pigs can be achieved [108]. Very recently, a nanoparticle-Poly(I:C) combination enhanced the immune response in pigs (high levels of virus neutralizing antibodies in bronchoalveolar lavage fluid) when compared to an inactivated IAV vaccine which was administered by the intranasal route and a multivalent commercial IAV vaccine given intramuscularly [109]. Of note, microscopic lung lesions and challenge virus loads were not different between vaccine groups [109].

### Assessment of vaccine efficacy

Vaccine efficacy is the percentage reduction of disease in a vaccinated group compared to an unvaccinated group, under the most favorable conditions and was originally defined in 1915 for human cholera and typhoid vaccines [110]. Commonly, vaccine efficacy is initially tested under experimental conditions using a defined challenge model in naïve pigs (negative for antibodies against the challenge virus). Parameters that may be assessed include reduction of clinical signs, macroscopic and microscopic lesions, amount and duration of pathogen shedding as well as impact on average daily weight gain compared to non-infected control pigs. In addition, reduction in transmission of the virus, speed and breadth of antibody responses to the vaccination may also be assessed among other parameters. Once experimental data are promising, field trials are initiated in large groups of pigs that may or may not have been exposed to the virus and hence may have active or passive immunity.

### Novel tools available to further improve vaccine development

The diagnosis and the characterization of pathogens have made much progress since DNA was discovered in 1869 by Friedrich Miescher [111]. The first attempts at sequencing DNA were performed in 1970 at Cornell University (https://web.archive.org/web/20090304121126/http://www.mbg.cornell.edu/faculty-staff/faculty/wu.cfm). Furthermore, genetic engineering, the designed and targeted manipulation of a genome, was first achieved in 1973 by Herbert Boyer and Stanley Cohen [112]. Detection of viruses is today commonly performed in veterinary diagnostic laboratories by polymerase chain reaction (PCR), to demonstrate their presence and quantify them. If further characterization of the virus is desired, this may be followed by subsequent sequencing which can often be conducted within 12–24 h after the request has been raised. Today, PCR and sequencing are considered basic diagnostic tools in veterinary diagnostic laboratories. In fact, at the Veterinary Diagnostic Laboratory at Iowa State University, Ames, USA, the number of PRRSV PCR tests for veterinary diagnostic purposes was 5232 in 2002 as compared to 60,565 in 2009 and 200,209 in 2019 (data courtesy of Dr. K. Harmon). Because of this rapid acceleration of knowledge, a variety of new and useful disciplines in vaccinology have evolved.

#### Reverse vaccinology

The inclusion of whole genome sequencing and bioinformatics in vaccine design, rather than relying only on serological evidence, was first introduced in 2000 [113]. Viable vaccine candidates are identified by computational methods, utilizing genomic information of virus specific B- or T-cell epitopes, signal peptides, or proteins with extracellular localization and are later validated by serological and immunological assays [114]. This approach allowed the identification of broadly cross-reactive T-cell epitopes from two pig IAV H1 lineages which were further verified by in-vivo analysis in infected pigs [115]. In another study, the extracellular domains in the ORF 5 gene of PRRSV were combined to create an engineered antigen which elicited neutralizing antibodies in vaccinated pigs [116].

#### Immunoinformatics

This approach allows understanding of immunological information through bioinformatics and computational approaches [117]. Analyses may be done at the level of epitopes, subunit vaccines, attenuated or inactivated vaccines. Goals of this approach are to efficiently and effectively predict immunogenicity and to identify antigens suitable for use in vaccines, characterizing MHC haplotypes [118, 119] and preferably demonstrating and confirming them in experimental challenge studies [87, 120]. It is expected that this approach will enable the discovery of more suitable epitopes and will overall accelerate vaccine design [121]. Pig vaccine applications to date have included viral infections such as IAV, PCV2, and ASFV [121]. Recently, this approach has also been utilized to predict epitopes and proteins for porcine rubulavirus [122].

#### Immunogenetics

The genetic basis of the immune response is studied using this discipline, including investigating normal immunological pathways and identifying variations that may result in immune response defects. For instance, IAV in-vitro refolding assays have been used to investigate the affinity and stability of the binding of epitope peptides to MHC class I molecules, which are key factors in the presentation of peptides to cytotoxic T lymphocytes [123]. Additionally, in the ongoing SARS-CoV-2 epidemic, understanding the immunogenetic factors influencing the immune response to infection is thought to be critical for patient management and identifying risk factors for fatal disease complications [124]. Similarly, understanding the host immunogenetic factors is important in designing veterinary vaccines for efficient control of viral infections.

#### Systems vaccinology

In this interdisciplinary approach, complex interactions among all the parts in a biological system are systemically described to ultimately predict its behavior [125]. Investigations may target viral antigens, virulence factors, viral host modulating genes, swine leucocyte antigen (SLA) polymorphisms, and pig immunogenetics, which directly and indirectly influence resistance to infection and development of immune responses. One potential application of this discipline is predicting vaccine efficacy against known or unknown strains of a certain pathogen [126]. Identification of molecular signatures, which are induced rapidly in the host after vaccination and correlate with the development of protective immune responses, represents a strategy to prospectively determine vaccine efficacy [126].

### Knowledge gaps

Although the production of vaccines against porcine viral diseases has progressed considerably, there are still many pig viral diseases with insufficient solutions. The swine industry would likely prefer to have single dose multivalent products that can be administered with minimal labour (i.e. via water, feed, air) and protect growing pigs from weaning to slaughter, by providing excellent efficacy against most variant strains that exist for a given virus. It should also be noted that for breeding herds and boar studs it would be desirable that products with zero risk of endangering the breeding stock are administered once a year (in contrast to the current need of administration every 6 months or at every reproductive cycle). Spray application/nebulization vaccination is commonly used in poultry, yet this technique has yet to be further developed for pig farms to make it effective, practical and cost effective. Experimental trials using intranasal nebulization for IAV vaccine administration have shown promising potential [127].

## Summary and discussion

The first veterinary vaccine for pigs became available approximately 150 years ago and today there is a large repertoire of pig vaccines available. Viral vaccines are used on a regular basis and are considered essential in modern pig production. With all the advances in recent years it remains puzzling why there is still no fully effective vaccine for some viruses, including PRRSV but also viruses that cause extremely high mortality such as ASFV. Induction of sterilizing immunity, i.e. complete protection against a virus, remains the goal, yet in reality no pig vaccine consistently induces sterilizing immunity and a low level of virus replication after active infection is not unexpected in vaccinated pigs. Interestingly, and along these lines, a US research team reported earlier this year on the development of an ASFV vaccine with sterilizing immunity against the current Eurasia pandemic strain [128]. This group was able to achieve complete attenuation of a highly virulent ASFV Georgia strain by deleting a previously uncharacterized gene, I177L [128]. We look forward to seeing how this product performs in the field and perhaps using this as a model for other pathogens.

Besides having a perfect/ideal vaccine against the target pathogen, important considerations for successful vaccination are the number of doses required, the vaccination route, and the correct timing as well as the general ease of vaccine administration. In fact, it is crucial in the early stages of vaccine development to clearly define the target product profile (TPP) [129]. To define the TPP, many things need to be considered including the vaccine’s target market, the importance of the disease (economical losses), number of doses used, thermostability and others. Mixing of vaccine types such as with heterologous prime boosting (for example one dose of an inactivated vaccine followed by a booster dose of an attenuated-live vaccine or a DNA vaccine) is gaining momentum in pig production [87, 130] as it may be possible to trigger a longer-lasting, broader immunity by using two different vaccine types for the same pathogen in the same pig.

In addition, novel viruses are discovered in pigs every year and these may or may not be associated with disease. Better and faster assessment of the importance of such viruses needs to take place. If a new virus or virus variant causes financial losses in pig farms, a vaccine needs to be available as soon as possible. This requires the simultaneous use of all tools available for pathogen assessment and subsequent vaccine design in several laboratories to attempt to reduce any possible losses for pig producers. Vaccine research would likely benefit from additional people working in the bioinformatics area cooperating with and complementing the work of traditional pathologists, virologists and immunologists. Much has improved since mass-vaccinations entered agriculture, but further improvements at the vaccination stage, such as avoiding the transmission of pathogens via vaccine equipment and reducing unnecessary stress to pigs also need to be considered in future vaccine developments.

## Conclusion

In this review of the literature, we summarized current information on traditional and novel methods to design, produce and administer viral vaccines for use in pig production. Although there has certainly been measurable progress since veterinary vaccines were introduced approximately 150 years ago, attenuated-live and inactivated vaccines are still most commonly used. With the advent of affordable and fast gene sequencing, genomic engineering, and bioinformatics we are likely soon to realize major advances in the type and efficacy of vaccines for pig producers with the opportunity to pick and choose a vaccine that is tailored to specific requirements.

## Data Availability

Not applicable. No data or materials were generated for this article.

## Abbreviations

ASFV: African swine fever virus
*B. subtilis*: *Bacillus subtilis*
CHO: Chinese hamster ovary cell line
CSFV: Classical swine fever virus
DIVA: Differentiation of infected from vaccinated animals
DNA: Deoxyribonucleic acid
*E. coli*: *Escherichia coli*
FMDV: Foot and mouth disease virus
HEK-293: Human embryonic kidney 293 cell line
HEV: Hepatitis E virus
IAV: Influenza A virus
IM: Intramuscular; M2e: Extracellular domain of the M2 antigen
MHC: Major histocompatibility complex
MVA: Modified vaccinia Ankara
ORF: Open reading frame
PCR: Polymerase chain reaction
PCV1: Porcine circovirus type 1
PCV2: Porcine circovirus type 2
PEDV: Porcine epidemic diarrhea virus
PRRSV: Porcine reproductive and respiratory syndrome virus
PRV: Pseudorabies virus
SAVE: Synthetic attenuated virus engineering
SARS-CoV-2: Severe acute respiratory syndrome-coronavirus-2
SUMO: Small ubiquitin-like modifier
TCID_50_: 50% tissue culture infectious dose
VLP: Virus-like particle
